# Synthetic Material for Bone, Periodontal, and Dental Tissue Regeneration: Where Are We Now, and Where Are We Heading Next?

**DOI:** 10.3390/ma14206123

**Published:** 2021-10-15

**Authors:** Chia Wei Cheah, Nisreen Mohammed Al-Namnam, May Nak Lau, Ghee Seong Lim, Renukanth Raman, Peter Fairbairn, Wei Cheong Ngeow

**Affiliations:** 1 Faculty of Dentistry, University of Malaya, Kuala Lumpur 50603, Malaysia; chiawei@um.edu.my (C.W.C.); minalau@um.edu.my (M.N.L.); g.s.lim@um.edu.my (G.S.L.); 2 School of Dental Sciences, Faculty of Medical Sciences, Newcastle University, Framlington Place, Newcastle upon Tyne NE2 4BW, UK; nis_moh2007@yahoo.com; 3 Oral Health Division, Ministry of Health Malaysia, Putrajaya 62590, Malaysia; extractions@gmail.com; 4 Department of Periodontology and Implant Dentistry, School of Dentistry, University of Detroit Mercy, 2700 Martin Luther King, Jr. Boulevard, Detroit, MI 48208, USA; peterdent66@aol.com

**Keywords:** bioglass, bone substitute, hydroxyapatite, polymers, synthetic

## Abstract

Alloplasts are synthetic, inorganic, biocompatible bone substitutes that function as defect fillers to repair skeletal defects. The acceptance of these substitutes by host tissues is determined by the pore diameter and the porosity and inter-connectivity. This narrative review appraises recent developments, characterization, and biological performance of different synthetic materials for bone, periodontal, and dental tissue regeneration. They include calcium phosphate cements and their variants β-tricalcium phosphate (β-TCP) ceramics and biphasic calcium phosphates (hydroxyapatite (HA) and β-TCP ceramics), calcium sulfate, bioactive glasses and polymer-based bone substitutes which include variants of polycaprolactone. In summary, the search for synthetic bone substitutes remains elusive with calcium compounds providing the best synthetic substitute. The combination of calcium sulphate and β-TCP provides improved handling of the materials, dispensing with the need for a traditional membrane in guided bone regeneration. Evidence is supportive of improved angiogenesis at the recipient sites. One such product, (EthOss^®^ Regeneration, Silesden, UK) has won numerous awards internationally as a commercial success. Bioglasses and polymers, which have been used as medical devices, are still in the experimental stage for dental application. Polycaprolactone-TCP, one of the products in this category is currently undergoing further randomized clinical trials as a 3D socket preservation filler. These aforementioned products may have vast potential for substituting human/animal-based bone grafts.

## 1. Introduction

Bone grafts and their substitutes are often necessary to provide support, fill voids and enhance biological repair of skeletal defects. Bone substitutes can be categorized into bone grafts (autograft, allograft, xenograft), ceramics/synthetics (hydroxyapatite, tricalcium phosphate, calcium sulphate) and growth factors (human demineralized bone matrix, platelet derivatives, bone morphogenic proteins [1]. A comprehensive review of commercially available bone grafts has been provided by Zhao et al. recently [2]. The objective of this current review is, therefore, to complement their report with regards to more recent updates on synthetic bone materials.

Whilst little advancement has been made with autograft, allograft and xenograft, recent discovery of permanent autogenous dentine deserves a brief mention [3,4,5,6,7]. Dentine has an excellent source of matrices, trace metal ions, and multiple growth factors, including TGF-β (transforming growth factor β), FGF-2 (fibroblast growth factor 2), and various angiogenic growth factors which are essential for bone tissue engineering [3,8,9,10]. The processed product, termed human demineralized dentine matrix (DDM) is an acid-soluble scaffold that contains a collagenous matrix and osteoinductive growth factors, in addition to a mineral phase. DDM-based scaffolds are reprocessed, acellular, and nanoporous. In addition, DDM can be used as a carrier of rhBMP-2 [11]. Studies have shown that dentine molecules function as regulatory signals for the healing and resorption of dental and periodontal tissues. They act as signaling and migratory stimuli for various mesenchymal and inflammatory cells [12].

Following gradual resorption, 46–74% of DDM are replaced with new bone through osteoinduction and osteoconduction in cases of guided bone regeneration (GBR), socket preservation, and ridge augmentation [13,14,15,16]. Koga et al. concluded that partially demineralized dentine matrix (PDDM) with large particles (1000 µm) has much more bone regenerative activity in comparison with undemineralized dentine (UDD) since demineralization enhances the osteoinductive capacity of tooth material by exposing organic substances within the dentine to the surface [6,14,17]. Nevertheless, some authors have reported successful bone regeneration applying UDD since non-demineralized particulate grafts are also osteoinductive [18].

Unlike permanent autogenous dentine, alloplasts are synthetic, inorganic, biocompatible bone substitutes that primarily function as defect fillers. In this case the material used functions as a bone filler, with the potential to upregulate host bone regeneration, but possibly lacking the quality of allografts and xenografts. In such instances, the bony defect may not form bone in its entirety. Besides biocompatibility, the acceptance of these substitutes by host tissues is determined by three important features—pore diameter, porosity and inter-connectivity. In addition, Palma et al. reported the influence of different formulations of bone grafts in providing an adequate scaffold, thus emphasizing the importance of the type of carrier in the three-dimensional distribution of particles and space provision in new bone formation [19]. This article appraises recent developments characterization, and biological performance of different synthetic materials for bone, periodontal, and dental tissue regeneration. They include calcium phosphate (CP) cements and their variants namely β-tricalcium phosphate (β-TCP) ceramics and biphasic calcium phosphates (BCP) (HA and β-TCP ceramics), calcium sulfate (CS), bioactive glasses (BG) and polymer-based bone substitutes.

## 2. Calcium Compounds

### 2.1. Calcium Phosphate Cements

One of the most promising groups of synthetic bone substitutes are calcium compounds, and among them are calcium phosphate (CP) and calcium sulphate (CS) cements. CP cement plays important roles in cell adhesion and tissue formation by affecting the adsorption of extracellular matrix proteins on their surface [20]. Calcium ions cause bone formation and maturation through calcification. It also affects bone regeneration through cellular signaling. Calcium stimulates mature bone cells through the formation of nitric oxide and induces precursor cells for bone tissue regeneration [21]. Calcium stimulates osteoblastic bone synthesis, increases the lifespan of osteoblast and regulates the formation and the resorptive function of osteoclasts [22]. Phosphate on the other hand, regulates the differentiation and growth of osteoblasts and the osteoblastic lineage. It also inhibits osteoclast differentiation and bone resorption by regulating the ratio of receptor activator of nuclear factor kappa-B ligand:osteoprotegerin (RANK-ligand:OPG) [23].

CP cement was first introduced as a synthetic bone substitute in the form of CP water setting cement by Brown and Chow 35 years ago [24]. In 1996, tetracalcium phosphate (TTCP) and dicalcium phosphate dihydrate (DCPD), the latter variants of CP cement, were approved by the United States (US) Food and Drug Administration (FDA) for the treatment of non-load-bearing bone defects [1]. CP exhibits mechanical properties such as high brittleness, low impact resistance, and low tensile stress due to the presence of pores [25,26]. It is used in the repair periodontal defects, augmentation of alveolar bone, sinus lifts, tooth replacement, and repair of large bone defects caused by tumors. It is also used as scaffolds in tissue engineering for bone or dentine regeneration. Traditionally, CP cement is contraindicated to substitute vascularized large bone defects as it lacks angiogenic properties. More recently, a new strontium–CP hybrid cement with enhanced osteogenic and angiogenic properties for vascularised bone regeneration has been introduced [27].

Depending on the ratio of calcium and phosphate, different variants of CP cement with slightly different physicochemical properties can be distinguished. CP cement can be classified into different groups with different stability and/or solubility: (1) HA and α-tricalcium phosphate (α-TCP); (2) BCP, and (3) DCPD and β-tricalcium phosphate (β-TCP) [28]. Table 1 lists the main calcium phosphate compounds used as bone substitutes and their Ca/P ratio [29] (Table 1).

Various new formulations of CP cement have been introduced, especially with bioinorganic supplementation such as strontium, magnesium, zinc or silicon to increase the biological performance of CP cement, especially in terms of bone regenerative potential. A recent meta-analysis of in-vitro studies reported that strontium, magnesium, and silica significantly enhanced new bone formation, while zinc did not have any effect. Moreover, strontium significantly enhanced and silicon inhibited CP cement degradation, while other bioinorganic supplementations generally did not promote material degradation at all [29].

Clinically, CP cement in various formulations consists of CP in white powder form, which when mixed with liquid, forms a workable paste. The paste can then be shaped according to the contour of bone to be substituted in the operating theatre or chairside. CP cement paste is easy to handle thus allowing it to be shaped to a complex bone cavity. Additionally, it can fill the space between the bone and the implant or any periodontal bony defect [30]. Some CP cements are available in injectable form and can thus be used in defects with limited accessibility or narrow cavities, via minimally invasive procedures, or tissue-sparing surgery with resultant lower costs and morbidity [31].

Once mixed, CP cement sets and hardens in 15 to 80 min depending on the formulation [31]. CP cements are biocompatible as the setting process is isothermal and does not change the physiological pH [1]. Once set, it forms nanocrystalline HA that is osteoconductive and bioresorbable up to 2 years without resorption, depending on its formulation [1,32]. Over time, new bone tissue regenerates to gradually replace the CP cement.

As stated earlier, CP cements are used to substitute and promote regeneration of non-load-bearing bone tissue as it is brittle. Thus, it is suitable for dental/oral and maxillofacial applications. The properties of CP cements affect bioactivity, such as adhesion, proliferation, and new bone formation in osteoblasts. Degradation and ion release in CP cements are bioactive features of this material [33]. A meta-analysis reported 13% of mean complication rate, with a wide variability ranging from 0% to 62%. Failure rate (9%) is rated the highest among these complications, followed by infection (5%). Other minor complications include the need for secondary surgery, the need for secondary contour revision, fragmentation, foreign body host reaction, and/or intractable seroma. It is contraindicated for surgical sites that are in communication with the paranasal sinuses or treatment combining radiotherapy due to a higher complication rate [34].

### 2.2. β-Tricalcium Phosphate Ceramic and Biphasic Calcium Phosphate

β-TCP and BCP are given special mention as they are two common formulations used in bone, periodontal and dental tissue regeneration. β-TCP ceramic allows fast bone tissue regeneration by human mesenchymal stem cells due to the interconnected porous structure that improves vascularization [35]. A coordinated action between osteoblasts and osteoclasts which lead to bone regeneration was reported in an animal model used to evaluate healing following implantation of β-TCP [36]. In addition, the degradation of β-TCP is compatible with the growth rate of newly formed bone [37]. It appears radio-opaque on radiographs thus improving the monitoring of healing. This material resorbs readily and presents with low immunogenicity [35]. It is suitable to be used in grafting of alveolar osseous defects [38], maxillary sinus floor grafting [39], and extraction socket grafting [40]. However, it has poor mechanical strength and is not suitable to be used in load-bearing areas [30].

BCP is a bone substitute which is a mixture of HA and β-TCP in fixed ratios. It combines the advantages of HA and β-TCP. BCP is obtained when a synthetic or biologic calcium-deficient apatite is sintered at temperatures at and above 700 °C. It has been used as scaffold for tissue engineering, drug-delivery system and carrier of growth factors [41]. Some of the dental applications are in maxillary sinus grafting [42] and extraction sockets grafting [43]. The properties of CP such as improved cell adhesion, proliferation, and new bone formation in osteoblasts can be enhanced when combined with the property of HA which resorbs slowly and serves as an effective scaffold for new bone formation. HA has excellent biocompatibility and bioactivity and can directly bond to the host bone [44]. In order to enhance the mechanical properties (brittleness) and bioactivity, CP ceramic scaffolds can be coated with biocompatible materials, while maintaining the macropores intact and open. Nanocomposite coatings consisting of poly-L-lactide (PLLA) as a polymer matrix and nanohydroxyapatite (HA) as ceramic filler were used to coat the BCP scaffolds [45]. The ratio of β-TCP/HA can be changed to achieve a desired degradation rate [46]. One well experimented ratio is the combination of 60% slowly resorbing hydroxyapatite (HA) and 40% fast resorbing beta-tricalcium phosphate (β-TCP) [47]. Table 2 provides a comparison between these two formulations (Table 2).

### 2.3. Calcium Sulphate and β-Tricalcium Phosphate

The other most promising calcium compound is CS, initially reported by Dreesmann [48], and has been in clinical use for more than 100 years. CS is a natural ceramic that is mined as gypsum (calcium sulfate dihydrate). Heating under controlled conditions produces the hemihydrate which exists in ß and α forms. The denser α-hemihydrate form (CaSO_4_·_0.5_H_2_O) is recommended for clinical use. This form is stronger and harder, which makes it more useful as a bone defect filler [49]. It has a compressive strength greater than that of cancellous bone [50] but undergoes resorption within a short period (3–6 weeks) [51,52] (Table 3).

CS is able to set and form a nano-porous cell occlusive membrane, preventing the early-stage invasion of unwanted soft tissue cells into the graft [50,53]. CS on its own is unable to bring about osteogenesis, but potentially encourages the formation of bone through its dissolution [54]. Consequences of CS dissolution include release of resorbable Ca^2+^ ions and localised acidity. The release of Ca^2+^ ions may help to stimulate osteoblasts and retard osteoclastic activity, whereas the localized acidity contributes to the antimicrobial feature of CS. However, CS alone is not an effective material as a bone filler since its resorption rate is significantly faster than bone growth, resulting in an absence of an appropriate scaffold within the defect. This means that CS has time-limited osteoconductive properties, as documented by many studies [52,55].

A recent study used combination of CS spheres with plasma rich in growth factors (PRGF) as a bone-graft substitute following removal of mandibular third molar [56]. The authors reported that the grafted sites showed significant bone regeneration compared to control sites. PRGF is autologous and is derived from the patient’s own blood through centrifugation to obtain concentrated growth factors from platelets [57]. In another study, the combination of CS with platelet-rich-fibrin (PRF) as a graft in extraction sites had similarly reported no significant linear and volumetric difference compared to sites grafted with a combination of xenograft and PRF [58]. PRF consists of growth factors in a fibrin network with gradual release into the environment thus maintaining the healing process [6,59]. Addition of CS not only promotes activation of plasma growth factors and accelerates bone formation, but also acts as a barrier to prevent epithelial downgrowth into the bony defect.

Although the CS and platelet concentrate composite holds great promise, a more common combination in clinical use is CS and CP. Apart from being osteoconductive, there is strong experimental evidence that CP also has osteoinductive properties [60,61]. CP ceramics in themselves seem to have the potential to influence angiogenesis [62]. The chemical changes induced by CP degradation and physical features of CP materials, such as porosity, influences new vessel formation. Chen and co-workers in 2014 demonstrated significantly up-regulated expressions of angiogenesis-related genes in the ingrowth of cells into the inner pores of CP ceramics [63]. All the results confirmed the angiogenic induction of porous CP ceramics, with β-TCP showing the highest potential. β-TCP is structurally porous and undergoes resorption over a 9- to 16-month period [64]. Unfortunately, there may be less bone volume produced than the volume of the graft material resorbed. For this reason, the clinical use of β-TCP has been rather as an adjunct with other less resorbable bone graft substitutes or as an expander for autogenous bone grafts [65,66].

The combination of CS and β-TCP enhances the handling properties of the graft material and produces a stable in situ hardening paste that adapts to the shape of bony defect, and at the same time is a porous bone substitute that serves as a scaffold for bone regeneration [67,68,69,70,71,72]. This is similarly seen in other forms of biphasic CS, where materials other than β-TCP are used [73]. From a clinical standpoint, a self-stabilizing graft reduces the need for membranes, resulting in shortened, less expensive and simplified surgical approaches. Without the foreign membrane, there is no impediment to the induction of stromal cell-derived factors by the periosteum. This is important as it results in the presence of mesenchymal cells at the bone healing site, which will differentiate into osteoblasts. Furthermore, the added stability may reduce micro-movements among graft particles and between the bone graft and the recipient site which may lead to the development of connective tissue instead of bone [74,75].

**Figure 1:**
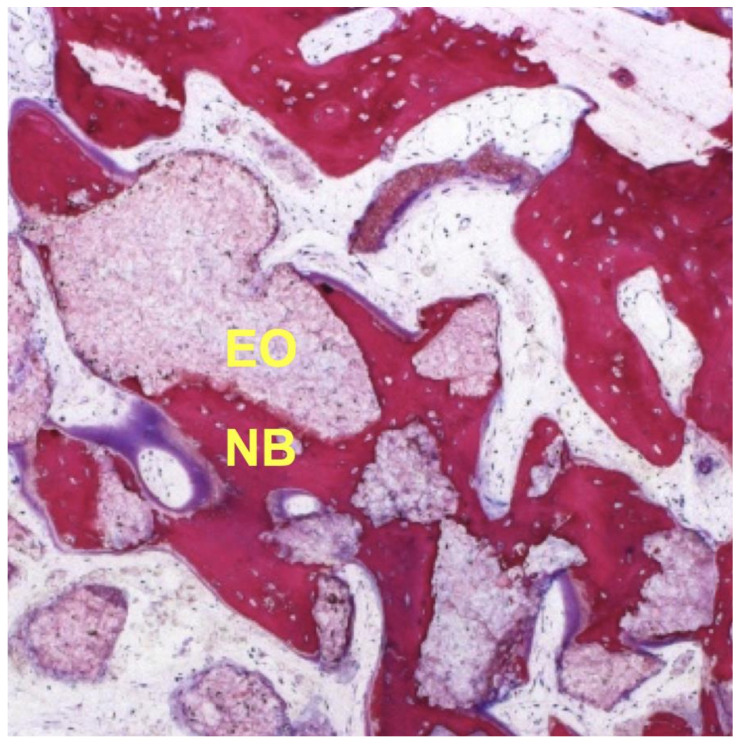
Histologic sections of EthOss^®^ grafted specimen. New bone (NB) 50.28% and residual EthOss^®^ (EO) 12.27% (H&E stain; X200).

The use of a composite graft containing CS and β-TCP has been described in several reports and studies [67,68,72,76,77,78,79], which demonstrated very favorable healing outcomes due primarily to improve access to the periosteal blood supply. The relatively fast resorption rate of CS allows for further neovascular ingrowth, with improved angiogenesis and up-regulated host healing. In contrast to conventional β-TCP, manufacturing and application of this biphasic calcium graft material uses a proprietary process to establish a negative zeta potential [79]. In an aqueous environment, the negatively charged graft surface is more accessible for the attachment and proliferation of osteoblasts when compared to an uncharged or positively charged surface. A study on the iliac crest of dogs by Podaropoulos et al. in 2009 [72] revealed that the mean percentage of new bone regeneration after 4 months by histological evaluation and morphometric analysis was 49.38%. Similar findings were reported in a multi-centre study by Fairbairn et al. in 2018. A bone core biopsy after 12 weeks of healing was obtained prior to implant placement. Histomorphometric analysis revealed that sites grafted with a Calcium Sulphate/β-TCP composite (EthOss^®^) was occupied by 50.28% of new bone, 12.27% of residual grafting material, and 37.45% of connective tissue [77] (Figure 1; Courtesy of Heiner Nagursky and Annnette Linder, Freiburg University, Tissue Department, Germany).

**Figure 2:**
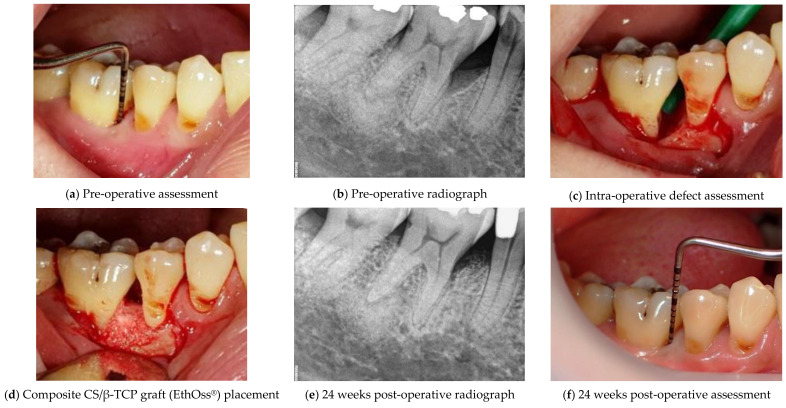
A clinical case showcases successful application of composite CS/β-TCP graft (EthOss^®^) to treat a periodontal defect.

Apart from alveolar ridge preservation, this novel composite graft material showed substantial promise in treating intrabony periodontal defects (Figure 2; Courtesy of Dr. Renukanth Raman). As shown, the combination of CS and β-TCP in EthOss^®^ provides an ease of use without the need of a membrane for guided bone regeneration. This combination has won numerous awards internationally including The Queen’s Award in the UK for its success in bone tissue regeneration.

A 12-month randomized controlled clinical trial by Stein et al. in 2009 concluded that the clinical outcomes of a composite CS and β-TCP graft were equivalent to autogenous bone, and superior to open flap debridement alone for the treatment of infrabony periodontal defects [79]. The use of the CS/β-TCP composite graft, however, enabled a less invasive surgical protocol. The main advantages for the choice of this material over the conventional membrane and graft technique to achieve periodontal regeneration are:

- Non-requirement of a membrane leading to reduced surgical time and cost;
- Self-stabilizing through hardening;
- Suitable resorption profile allowing cell occlusive properties, adequate porosity, volume maintenance and high rate of turnover to new host bone.

## 3. Bioactive Glasses

Bioactive glasses (BG) are a group of synthetic alloplastic reactive materials, with a silicate base that has the unique ability to form bonding with mineralized hard tissue such as bone in a physiological environment. BG was first developed by Larry Hench and his colleagues in 1969 [80,81]. The idea of BG was based on a simple hypothesis: “As hard tissues such as bone contain a hydrated calcium phosphate component, namely hydroxyapatite (HA), any material with the ability to form a HA layer in vivo may not be rejected by the body” [82].

The mid-1980s marked a milestone for the application of BG in dentistry when Clark, Stanley and Hall successfully applied BG in a clinical trial for alveolar ridge preservation on edentulous patients. The results of the clinical trial paved the way for legal approval of Bioglass^®^ for commercial use in the United States of America. However, it was not until the 1990s that the BG was first applied in periodontal lesions [83,84]. Since then, the pioneer brand, Bioglasss^®^/45S5 and several new modifications have been extensively studied in various fields of dentistry [82,84].

The first-generation BG consists of silicon dioxide (SiO_2_), sodium oxide (Na_2_O), calcium oxide (CaO), and phosphorus pentoxide (P_2_O_5_). These first-generation BGs (e.g., Bioglass^®^ 45S5, S53P4) come in various forms (dense or particulate) with various clinical applications. However, these BG have a high tendency to form crystals during manufacturing under high temperature to form various shapes and forms. To overcome this problem, additives such as boric anhydride, potassium oxide, and/or magnesium oxide have been added to the original formula, thus developing the novel approach [85] that is able to produce BG in various useful forms such as microspheres, fibers, and porous implants [81].

The second-generation BG has a unique composition range of calcium sodium phosphosilicate (CSPS) glasses and glass-ceramics which enable BG to promote bonding between implant material and bone [86]. On the other hand, the third-generation BG has the ability to release biochemical stimuli to cater for the increasing inclination towards a more biological-based approach to regeneration of the diseased or damaged hard tissues [86]. When nanotechnology came into play, the particle size of BG reduced from micro to nano scale and thus increased the surface area for HA formation in a shorter time which greatly improved the bioactivity of BG [84].

Generally, the rate of the bioactivity depends on not only the surface area, but also the chemical composition of the BG. The crucial determining factor is the SiO_2_ content that has to be <60% in weight, ideally. It was found that BG containing 45–52% of SiO_2_ in weight produced BG with the most rapid bonding [84,87]. BG can be categorized based on its composition and processing method, as shown in Table 4.

When BGs are in contact with body fluid, two stages of bioactivity are initiated, the chemical exchange mechanism involving hydroxycarbonate apatite layer (HCA) formation on the BG, and the cellular mechanism involving osteogenesis [88]. There will be rapid ion exchange of sodium and potassium from the BG with the cation (H^+^) from the body fluid to form the silanol bond (Si–OH). The accumulation of this silanol group will increase the surrounding pH and promote further chemical attack to the silica glass network of BG to release more of the silanol group (–OH). The silanol group will then undergo condensation and form a rich silicone layer. The calcium and phosphate will then migrate from the extracellular fluids onto the silicone-rich layer [81,88].

Subsequent to the aforementioned chemical exchange stage, a HCA layer that is suitable for bone formation is formed. Bone-forming progenitor cells and other related cells will attach to it and differentiate to form a bone matrix. To date, the detail of the stages remains unclear. Nevertheless, numerous in vitro studies reported superiority of BG over other materials in providing surfaces for the attachment of the osteogenic cells for bone formation [88,89].

The first commercial clinical application of BG was middle ear bone replacement for treatment of conductive hearing loss which was first marketed in 1985 [82]. It is a type of monolithic medical device employing Bioglasss^®^/45S5 [88]. Since then, BGs have been manufactured in various sizes, shapes, and forms such as in granules, bulks, powder form, scaffold, and recently, in nanoparticles. Their usage in the medical and dental fields ranges from solid medical devices, bone regenerations in oral and maxillofacial bony defects, bone graft fillers, coatings on implants, to oral care products to treat dentine hypersensitivity [88].

## 4. Polymer-Based Bone Substitutes

Polymers are relatively large molecules composed of repeating monomers combined by covalent bonds [90]. In clinical sciences, they are considered very good choices for bone tissue engineering since they possess an exceptional flexibility, and adjustable characteristics through manipulating their chemical compositions. These characteristics precisely provide a window of opportunity to regulate the scaffold properties such as biocompatibility, mechanical properties, porosity, biodegradation topography and wettability that directly affect the bone regeneration efficiency [91]. They can be shaped in different forms or 3D printed into the desired 3D scaffold or can be made available as an injectable form [92].

Based on their source, polymer-based bone substitutes can be classified as natural polymer or synthetic (manufactured) polymer which in turn are categorised into non-biodegradable such as poly(methyl methacrylate) (PMMA) or biodegradable such as those derived from natural raw materials or synthesized from monomers derived from petroleum feedstock through a chemical reaction [93]. They have been found to be valuable in oral and maxillofacial areas for dental implants placement as a bone substitute, periodontal regeneration as a membrane in guided tissue regeneration and as a mesh for craniofacial reconstruction. The advantage shown by these materials is that of better osseointegration since they also aid in deposition, precipitation, and enhancing the formation of bone matrix [41].

**A. Development**

Polymer-based biomaterials were used in the human body soon after the invention of synthetic polymers. In 1960, Charnley introduced self-polymerizing PMMA cement for connecting the femoral head prosthesis to femur shaft [94]. It showed exceptional primary fixation but could not simulate a biological secondary fixation to the prosthesis due to its inactive nature. One study in 2008, reported that the PMMA inhibited the newly formed bone rather than promoted it [95].

The second-generation biomaterials involved naturally and synthetically derived biodegradable polymers [96]. Natural biodegradable polymers include proteins (collagen, silk fibroin, fibrinogen) or polysaccharides (chitosan, alginate, hyaluronic acid derivatives). Because of their biocompatibility, bioactivity, and biodegradability, they have been significantly studied as bone defect repair material or bone graft substitutes. In the earliest work of cell encapsulation, they have been used as hydrogels showing effective findings in non-load bearing areas [97,98]. However, their source instability, fast degradation rate, weak mechanical strength, and possible immunogenicity and denaturation during processing made them less effective when used alone [99]. On the other hand, biodegradable synthetic polymers received interest in bone regeneration because of their availability and low cost in comparison to the natural polymers. They showed controllable biological physicochemical properties than natural polymers; however they lack sufficient biological recognition signals [100]. Among them, synthetic aliphatic polyesters have been extensively studied because of their biocompatibility, degradability and their controllable design, mechanical and physical properties, which is vital for bone regeneration in various clinical applications. The main disadvantage of these types of polymer lies with their degradation products that might cause a reduction in the local pH. This not only accelerates the degradation process but also induces a local inflammatory reaction [101]. Later on, both natural and synthetic polymers were combined with each other or with other synthetic biomaterials such as ceramics to provide an advantage over other biomaterials [102,103]. For example, addition of hydroxyapatite to collagen sheets provided higher stability and resistance and improved the wettability [104].

Third-generation biomaterials were planned to integrate bioactive molecules into the polymer to induce cell differentiation, proliferation and bioactivity [105]. Recently, polymer biomaterials have been mostly used as a composite rather than used alone in bone-replacement applications due to their immunogenic reaction and weak mechanical strength. They are blended with each other or other inorganic materials such as ceramics to meet the specific needs for bone regeneration [106]. They can be encapsulated with bioactive molecules or growth factors to achieve controlled delivery of these molecules that have the ability to promote osteoblast differentiation and increase bone formation [107]. Currently, biodegradable polyesters are combined with ceramic such as HA and growth factors to be used as bioink for creating a 3D scaffold [108].

This section will focus on the most used synthetic polymer-based bone substitute polymers. In line with the previous studies, the most used polymer-based bone substitute with their merits and demerits are described in Table 5.

**B. Synthetic polymer biomaterials**

Synthetic polymer-based bone substitutes can be further classified as non-biodegradable such as PMMA, and biodegradable such as aliphatic polyesters’ families. According to the chemical structure, the aliphatic polyesters are introduced as either hydrophilic like PGA or hydrophobic like PLA. Their degradation rate and mechanical properties can be modified by altering their chemical composition and post-polymerization time [109]. The polyester was observed to be elastomeric and cyto-compatible in vitro but elicited a slight immune response in vivo. Furthermore, it was found that higher intrinsic viscosity and crystallinity (less space for H_2_O to access) is associated with longer degradation time, whereas higher porosity and surface area availability (facilitate the H_2_O to access) is associated with faster resorption time [110]. It demonstrates that with the gradual degradation of synthetic polymer, accompanying decreased mechanical properties, the load will gradually be transferred from the graft material to human bones and soft tissue to avoid a stress shield effect (stress stimulation to the new bone is gradually strengthened), which can help in promoting bone formation and remodeling.

In terms of their medical application, they have been employed for the production of various medical devices, such as plates, bone-fixation devices, sutures, stents and screws and in controlled drug-delivery vehicles [111]. In bone regeneration, the polymers are usually formed as coatings on biomedical devices, or in the form of as micro- and nanospheres for targeted drug delivery or composite with other inorganic biomaterials. This holds promises in periodontal therapy and regenerative medicine.

The most aliphatic conventional polyesters manufactured by fused deposition modelling (FDM) technology and used in medicine are polycaprolactone (PCL), polylactic acid (PLA), polyglycolic acid (PGA), and their copolymer poly(lactic-co-glycolic) acid (PLGA) [112].

### 4.1. Poly(Methyl Methacrylate) (PMMA)

PMMA is a rigid hydrophobic thermoplastic polymer, produced by polymerization of methyl methacrylate through a mass, emulsion, or solution polymerization process [113]. Despite the disadvantages mentioned above, it is still used clinically with similar success rate as bone cement which is formed through a mass polymerization reaction of methyl methacrylate (MMA) via free radicals, where their reaction products can induce local inflammation. Effectively, it is possible to make the properties of PMMA-based bone cements more tissue-friendly by adding 10% of vitamin E as an antioxidant which could reduce the number of free radicals formed. In addition, a functional active composite structure of PMMA that is bioactive and porous could be created by slightly changing its content such as the addition of bioactive material (bioactive glass) to its matrix.

### 4.2. Polycaprolactone (PCL)

PCL is a semicrystalline thermoplastic polymer with a slow degradation rate that maintains its mechanical feature. It is synthesised by ring-opening polymerisation to give thermoplastic elastomers with lower melting temperature (60 °C). It has been studied due to their microstructure being similar to the trabecular bone and its activity to encourage vascularization and cell communication [114]. It is considered less costly compared to the other polyesters such as PLA, PGA, and their copolymers, however, it has higher hydrophobicity and crystallinity, and a slower degradation rate than the others. To overcome these disadvantages, surface modification such as loading of bioactive molecules and plasma treatment are usually employed. Wang et al., found the addition of pristine graphene to PCL via FDM has a positive impact on cell viability and proliferation [115]. PCL polymer membrane contributed to the early biodegradation of β-TCP without affecting the bone regeneration capacity in a canine mandibular defect [116].

Furthermore, PCL could be copolymerized with other monomers to take on different functional groups, which can be further modified to enhance its bioactivity [110]. It can be combined with other biomaterials such as gelatin to enhance cell adhesion, proliferation and to accelerate its biodegradation rate [117]. A novel material named poly(caprolactone trifumarate)-gelatin microparticles (PCLTF-GMPS) was created using different ratios of PCL, Poly(propylene fumarate) (PPF) and gelatin. The biocompatibility and osteoconductivity of the created scaffold was demonstrated through the bone deposition and complete healing in critical size cranial defects in a rabbits [118]. Additionally, it revealed that the scaffold’s mechanical strength can be increased by boosting the ratio of PCL content in a composite.

Another option is the incorporation of TCP into the PCL. In recent years, a novel hybrid scaffold composed of a three-dimensionally (3D) printed polycaprolactone (PCL) HA/β-TCP scaffold was developed with simultaneous implant fixtures use in mind [119]. Clinically, another novel 3D printed PCL-TCP (Osteo-plug^®^; Ostepore International, Singapore) device for ridge preservation has been tested and is in the midst of undergoing further clinical trials. It is shown to have high porosity and bioactivity that promotes osteogenesis and reduce resorption while leveraging its 3D shape to fit snugly in the tooth socket [120]. In dentistry, PCL alone has been shown to provoke differentiation, colonization, proliferation of odontogenic human dental pulp cells isolated from mature teeth into functional odontoblast-like cells within the PCL cone, secreting extracellular matrix similar to the mineralized dentine matrix [121].

### 4.3. Poly(Lactic Acid) (PLA), Polyglycolic Acid (PGA), and Poly(Lacticco-Glycolic Acid) (PLGA)

PLA, PGA, and their co-polymer (PLGA) are available in different shapes from mesh for orthopaedic applications to drug eluting coatings on vascular stents.

PLA is a biodegradable thermoplastic polyester developed by polymerization of chiral semicrystalline molecules named D- and L-isomer [122]. The L-form shows a high crystallinity with high strength and long degradation time. Because of the poor surface on neat PLA surfaces compared to ceramic biomaterial, it is combined with other biomaterials. Currently, most 3D printed scaffolds use PLA and PLGA to create composite with other inorganic material to produce customized substitutes. In an experimental study, 3D maxillary sinus model was fabricated using the composite material from osteogenic HA-PLA [123].

PGA is an aliphatic polyester that has a regular linear molecular structure with exceptional tensile module, controlled solubility, and a high degradation rate. The degradation product of the PGA, glycolic acid, is excreted in urine. Clinically, it has been used as the first biodegradable suture for many years. Compared to other polyester such as PCL and PLA, PGA has a higher mechanical strength. Nonetheless, it is not suitable to be used alone for bone repair because of its high degradation rate in vivo. Three-dimensional porous composite scaffolds of PGA/β-TCP (in 1:1 ratio) showed a strong ability to regenerate bone with a degradation rate of 90 days [124]. In dentistry, a novel co-polyester, poly (butylene succinate-coglycolate) (PBSGL), has been fabricated by electrospinning in an attempt to produce a membrane for guided tissue regeneration in periodontology. It is reported that a higher ratio of PGA in PBSGL membranes resulted in better cell attachment and metabolic activity, additionally, and improved osteogenic potential with no adverse inflammatory response [125].

PLGA is a linear copolymer of lactic acid and glycolic acid monomers formed by the ring-opening polymerization of PLA and PGA. The performance, mechanical properties and its degradation rate could be adjusted by the different ratios of these two polymers. It has been reported that the scaffold of PLGA with the lactic acid and glycolic acid ratios of 75/25, respectively, have approximately half the degradation rate of scaffold with the ratio of 85/15 [126]. Like other polyesters, PLGA scaffolds have been used as carriers. Recently, various bioactive molecules have been loaded with PLGA/HA scaffolds to aid in bone healing. It has been demonstrated that nano-HA could improve the bone repairability of scaffolds [127].

In dentistry, Ohara et al. showed hard tissue formation in the back of mice after implantation of porcine tooth germ-derived cells with PGA fibre and β-TCP scaffolds [128]. Moreover, Thomas et al. investigated the role of PLA socket space fillers created by fusing porous PLA particles loaded with antibiotic solutions. They indicated that biodegradable drug-releasing polylactide space fillers could help to promote bone regeneration, and had the potential to be used for ridge preservation [129]. These results were also reported in alveolar sockets grafting procedures by Serino et al., who confirmed clinically and histologically that the bone resorption following tooth extraction is reduced by using a polylactide-polyglycolide acid bioresorbable synthetic sponge [130]. PLGA in combination with stromal cells from the adipose tissue demonstrated an ability to repair periodontal defects [131]. Furthermore, platelet-derived growth factor delivery by poly-(d,l-lactide) and poly-(d,l-lactide-co-glycolide) (PDLLA-PLGA) microspheres showed the ability to accelerate osteogenesis, bone maturation, fibres re-alignment, and cementogenesis in grafting periodontal bony defects [132].

## 5. Discussion and Conclusions

The search for synthetic bone substitutes remains elusive. This narrative review provides an update that complements an earlier publication. The authors have intentionally left out any materials that are of human/animal or organic origin as the objective is to concentrate solely on synthetic material. Hence, some composite material incorporating synthetic and organic material e.g., growth factors, are only mentioned briefly, at best. The future perspective of synthetic materials were reviewed thoroughly by Genova et al. in 2020, and we agree that the future direction is 3D printing [133]. Based on the materials reviewed, calcium compounds (CP, CS, β-TCP and HA in different formulation) currently provide the best synthetic substitute for dental use. Among them, the composite of calcium sulphate and β-TCP provides the most ease of use without the need of a membrane for guided bone regeneration. Another composite, polycaprolactone-TCP, is worth a mention as it is currently undergoing further randomized clinical trial as a 3D socket preservation filler, heeding the call of Zhao et al. that more materials should be tested in this manner prior to commercialization [2]. This process will heed the concerns of safety and efficacy of newer synthetic materials which are often by-passed after animal studies.

## Figures and Tables

**Table 1:** Main calcium phosphate (CP) compounds used as bone substitutes and their Ca/P ratio [29].

Compound	Chemical Formula	Ca/P Ratio
Dicalcium phosphate anhydrous (DCPA)	CaHPO_4_	1
Dicalcium phosphate dihydrate (DCPD)	CaHPO_4_·H_2_O	1
Amorphous calcium phosphate (ACP)	CaxHy(PO_4_)z·nH_2_O n = 3–4.5; 15–20% H_2_O	1.2–2.2
α-Tricalcium phosphate (α-TCP)	α-Ca_3_(PO_4_)_2_	1.5
β-Tricalcium phosphate (β-TCP)	β-Ca_3_(PO_4_)_2_	1.5
Calcium deficient hydroxyapatite (CDHA)	Ca_10_−x(HPO_4_)x(PO_4_)_6_−x(OH)_2_−x	1.5–1.67
Hydroxyapatite (HA)	Ca_10_(PO_4_)_6_(OH)_2_	1.67
Tetracalcium phosphate (TTCP)	CaO·Ca_3_(PO_4_)_2_	2.0

**Table 2:** Comparison between β-tricalcium phosphate (β-TCP) ceramic and biphasic calcium phosphate (BCP) [2].

Material	Advantage	Disadvantage	Indication/Application
Beta tricalcium phosphate (β-TCP) (i.e., IngeniOs™ Zimmer Biomet Dental, Carlsbad, CA, USA; Cerasorb™ Zimmer Biomet Dental, Carlsbad, CA, USA; OSferion^TM^ Olympus terumo biomaterials coorperation, Sasazuka, Japan; Orthograft^TM^ DePuy, Chester County, PA, USA)	Ease of handling Radiopacity allowing monitoring of healing Resorb readily Low immunogenicity Compressive strength similar to cancellous bone	Poor mechanical properties in particular compressive strength	Filler for alveolar defects (periodontal, periapical surgery, peri-implant and cyst enucleation) Extraction sockets grafting Sinus floor elevation
Biphasic calcium phosphate (i.e., Mastergraft^TM^ Medtronic, Minneapolis, MN, USA; Maxresorb^®^ Botiss dental, Berlin, Germany) * HA/β-TCP = 40/60	Resorb readily Greater mechanical strengths than either TCP or HA alone	Compressive strength remains lower than that of cortical bone	Filler for alveolar, periodontal and cystic defects Extraction sockets grafting Ridge augmentation Sinus floor elevation Periapical surgery

* indicating ratio of hydroxyapatite (HA) to β -tricalcium phosphate (β-TCP).

**Table 3:** Mechanical properties of calcium sulphate (CS) versus native bone.

Typical Mechanical Properties	Wet CS	Dry CS	Cancellous Bone	Cortical Bone
Compressive strength (MPa)	10–15	20–30	5–10	162.2
Tensile Strength (MPa)	2–4	4–6	10–15	151.8

Reproduced with permission from Elsevier (Ricci, J.L., Weiner, M.J. et al. Calcium Sulphate: Bioceramics and their Clinical Application. 2008.

**Table 4:** Categorization of bioactive glasses.

**Bioactive glasses**	Composition	Borate
		Phosphate
	Method of processing	Melt-derived
		Sol-gel

**Table 5:** The most commonly used polymer-based bone substitute.

Polymer Bone-Based Material	Type	Advantages	Disadvantage	Applications
Non-biodegradable				
**Poly methyl methacrylate (PMMA)**	Acrylic glass	Biocompatible, biologically inert durable, superior osseointegration	Non-degradable, residual monomer can enter the bloodstream and cause embolism, limited biological response, shrink during polymerization leading to free spaces between cement and prosthesis or bone, excess of tension can cause cement fractures and release of cement particles caused inflammatory reaction, exothermic polymerization	Orthopaedic prostheses fixation, craniofacial defects, dentures, vertebroplasty and kyphoplasty
Biodegradable				
**Poly(lactic acid) (PLA)**	Aliphatic polyester.	Biodegradable, tunable physical and mechanical properties. osteoconductive, biodegradable, biocompatible, promote bone regeneration, Crystallinity tunable by changing hydroxylation degree	Acidic degradation products that might cause adverse tissue reactions, and lack of cellular adhesion due to hydrophobicity	Orthopaedic fixation tools, tendon and ligament repair, vascular stents, bone graft extender, carriers of bioactive factors. PLA, PLGA block copolymers use for drug eluting coatings
**Poly(glycolic acid) (PGA)**				
**Poly(lactic-co-glycolic acid) (PLGA)**				Act as a copolymer of PLA and PGA, similar application spectrum as PLA
**Poly** **caprolactone (PCL)**		Biodegradable, Machinability, good mechanical strength, high porosity, crystallinity and thermal stability, crosslink in situ, printed by injection	Slow degradation rate, poor water wettability, lack of cell adhesion, low mechanical strength	Production of specialty polyurethanes, composite with other biomaterial to create tissue-engineered scaffolds, injectable implants for controlled release drug-delivery systems
**Poly** **(vinyl alcohol) (PVA)**	Polyalcohol	Biodegradable, tunable water solubility and crystallinity, biocompatible	Lower water solubility and crystallinity, cross-linking of polymers to maintain integrity	Used in tissue-engineering applications from the laboratory to the pre-clinical research
**Poly** **(propylene fumarate) (PPF)**	Unsaturated linear polyester	Osteoconductive, biocompatible, tunable degradation time, controllable mechanical properties, double bond along its backbone permits cross-linking in situ	Cross-linking of polymers to maintain integrity	Holds promise for use as regenerative scaffolds and bone cements often as part of an injectable bone replacement composite

## Data Availability

The data presented in this study are available on request from the corresponding author.
